# Genetic and Cellular Characterization of *Caenorhabditis elegans* Mutants Abnormal in the Regulation of Many Phase II Enzymes

**DOI:** 10.1371/journal.pone.0011194

**Published:** 2010-06-17

**Authors:** Koichi Hasegawa, Johji Miwa

**Affiliations:** College of Bioscience and Biotechnology, Chubu University, Kasugai, Aichi, Japan; University of Missouri-Kansas City, United States of America

## Abstract

**Background:**

The phase II detoxification enzymes execute a major protective role against xenobiotics as well as endogenous toxicants. To understand how xenobiotics regulate phase II enzyme expression, acrylamide was selected as a model xenobiotic chemical, as it induces a large number and a variety of phase II enzymes, including numerous glutathione S-transferases (GSTs) in *Caenorhabditis elegans*.

**Methodology/Principal Findings:**

To begin dissecting genetically xenobiotics response pathways (*xrep*), 24 independent mutants of *C*. *elegans* that exhibited abnormal GST expression or regulation against acrylamide were isolated by screening about 3.5×10^5^ genomes of *gst::gfp* transgenic strains mutagenized with ethyl methanesulfonate (EMS). Complementation testing assigned the mutants to four different genes, named *xrep-1*, *-2*, *-3*, and *-4*. One of the genes, *xrep-1*, encodes WDR-23, a nematode homologue of WD repeat-containing protein WDR23. Loss-of-function mutations in *xrep-1* mutants resulted in constitutive expression of many GSTs and other phase II enzymes in the absence of acrylamide, and the wild-type *xrep-1* allele carried on a DNA construct successfully cured the mutant phenotype of the constitutive enzyme expression.

**Conclusions/Significance:**

Genetic and cellular characterization of *xrep-1* mutants suggest that a large number of GSTs and other phase II enzymes induced by acrylamide are under negative regulation by XREP-1 (WDR-23), which is likely to be a functional equivalent of mammalian Keap1 and a regulator of SKN-1, a *C. elegans* analogue of cap-n-collar Nrf2 (nuclear factor erythroid 2-related factor 2).

## Introduction

Xenobiotics such as harmful food substances (e.g., acrylamide and 2-amino-1-methyl-6-phenylimidazol [4,5-*b*] pyridine), environmental pollutants (e.g., heavy metals like mercury and cadmium), or mycotoxins and exotoxins from contaminating microorganisms are direct or indirect threats that incur mutagenic, carcinogenic, teratogenic, endocrine disruptive, or other deleterious consequences [1]–[5]. Oxidative processes, such as oxidative phosphorylation indispensable for aerobic organisms to produce ATP via respiration, ironically produce highly active free radicals in the process: these radicals are thought to contribute to cancer, atherosclerosis, inflammation, hypertension, and diabetes [6]. Against all such exogenous and endogenous toxicants, phase II enzymes, with glutathione S-transferases (GSTs) as most prominent, are considered to play a major protective role [7]–[11]. As for GSTs, we should not dismiss other critical biochemical roles some GSTs play in such processes as eicosanoid or steroid hormone biosynthesis and amino acid metabolism [9], [12], [13].

Acrylamide, now recognized as a prevalent food substance, has been long known as a neurotoxin for many animals and a potential carcinogen for humans [1], [14]. Previously we reported in the nematode *Caenorhabditis elegans* [11] that acrylamide up-regulates a large number of phase II enzymes such as GSTs and UDP-glucuronosyl/glucosyl transferases (UGTs) and some phase I enzymes such as short-chain type dehydrogenases (SDRs). *C. elegans* offers many experimental advantages as a model for understanding diverse aspects of biology, including responses to xenobiotics [15]. In mammals, acrylamide might be detoxified mainly by GSTs and excreted in urine [16], [17]. Among GSTs that acrylamide up-regulated more than two-fold, those we studied displayed spatially varied expression patterns. For all these reasons we have selected acrylamide as a representative chemical for xenobiotics exposure.

To dissect genetically a xenobiotics response pathway (*xrep*) from a target of xenobiotics to the final destination of phase II enzyme expression, we isolated *xrep* gene mutants with abnormal GST expression or response to acrylamide. Here we report on one of four genes defined by these mutants, *xrep-1*, that encodes a WD repeat-containing protein, a nematode homologue of mammalian WDR23, and provide genetic and cellular evidence that the gene *xrep-1* negatively regulates GSTs and some other phase II enzymes.

## Results

### Isolation and mapping of mutants showing abnormal GST regulation

**Figure 1:**
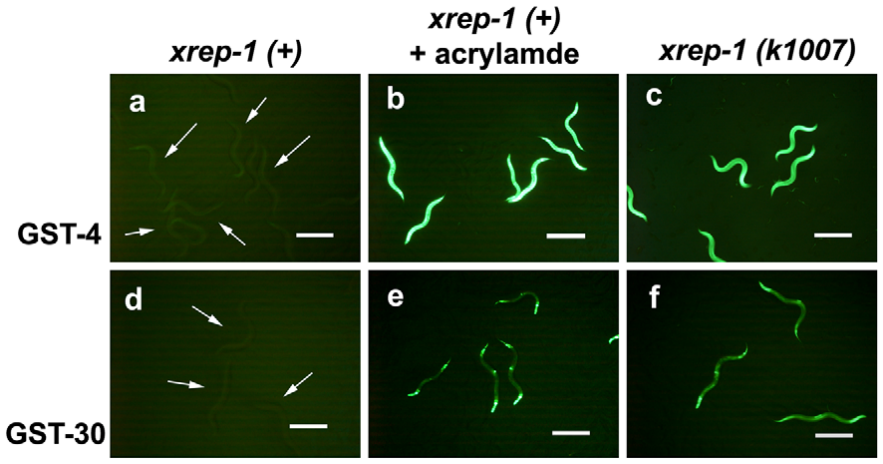
The *xrep-1(k1007)* mutants constitutively express GST-4 and GST-30 in the absence of acrylamide. (a) MJCU017 wild-type *xrep-1(+)* animals. Without acrylamide, no GST-4::GFP expression is detected (arrows). (b) MJCU017 wild-type *xrep-1(+)* animals treated with 500 mg/L acrylamide for 24 hours at 20°C. GST-4::GFP expression is induced. (c) The GST-4::GFP expression pattern in the *xrep-1(k1007)* mutants. Without acrylamide, a GST signal is detected from the whole body. (d) MJCU047 wild-type *xrep-1(+)* animals. Without acrylamide, no GST-30::GFP expression is detected (arrows). (e) MJCU047 wild-type *xrep-1(+)* animals treated with 500 mg/L acrylamide for 24 hours at 20°C. GST-30::GFP expression is observed in the pharynx, hypodermis, and intestine. (f) The GST-30::GFP expression pattern in the *xrep-1(k1007)* mutants. GST-30::GFP expression is detected from the pharynx, hypodermis, and intestine. Scale bars, 500 µm.

We used two *gst::gfp* transgenic animals, MJCU017 and MJCU047, to screen for mutants abnormally expressing GFP. MJCU017 and MJCU047, which have the chromosomally-integrated *gst-4::gfp* and *gst-30::gfp* fusion genes, respectively, emitted no detectable GFP signal in the absence of acrylamide, but emitted a very strong GFP signal from the whole body when treated with acrylamide (Fig. 1) [11], [18]. Mapping with a set of conventional marker mutants located both of these fusion genes as being integrated in linkage group X (data not shown). These animals were outcrossed at least three times with the wild-type strain N2.

We mutagenized these transgenic strains with ethyl methanesulfonate (EMS) and screened about 3.5×10^5^ genomes to obtain 24 independent mutants. The mutants were then outcrossed more than three times with N2 wild type to reduce unwanted mutations. Complementation testing and linkage analysis assigned 16 of the mutations defined by these 24 mutants to the same gene on linkage group (LG) I (chromosome I): all of the 16 mutations were recessive, and the affected strains constitutively expressed GST in the whole body without acrylamide. Six, assigned to the same gene on LG II, were also recessive, and their mutants constitutively expressed GST in the body-wall muscle and pharynx only after they had reached the adult stage. Of the remaining two strains, one mutation on LG IV was dominant, with constitutively expressed GST throughout the whole body, whereas the other one, on LG I, was recessive with its mutant expressing no GST even when treated with acrylamide. We called these genes defined by the four complementation groups *xrep-1*, *-2*, *-3*, and *-4*, in the respective order described above.

### The *xrep-1* gene is *wdr-23*

**Figure 2:**
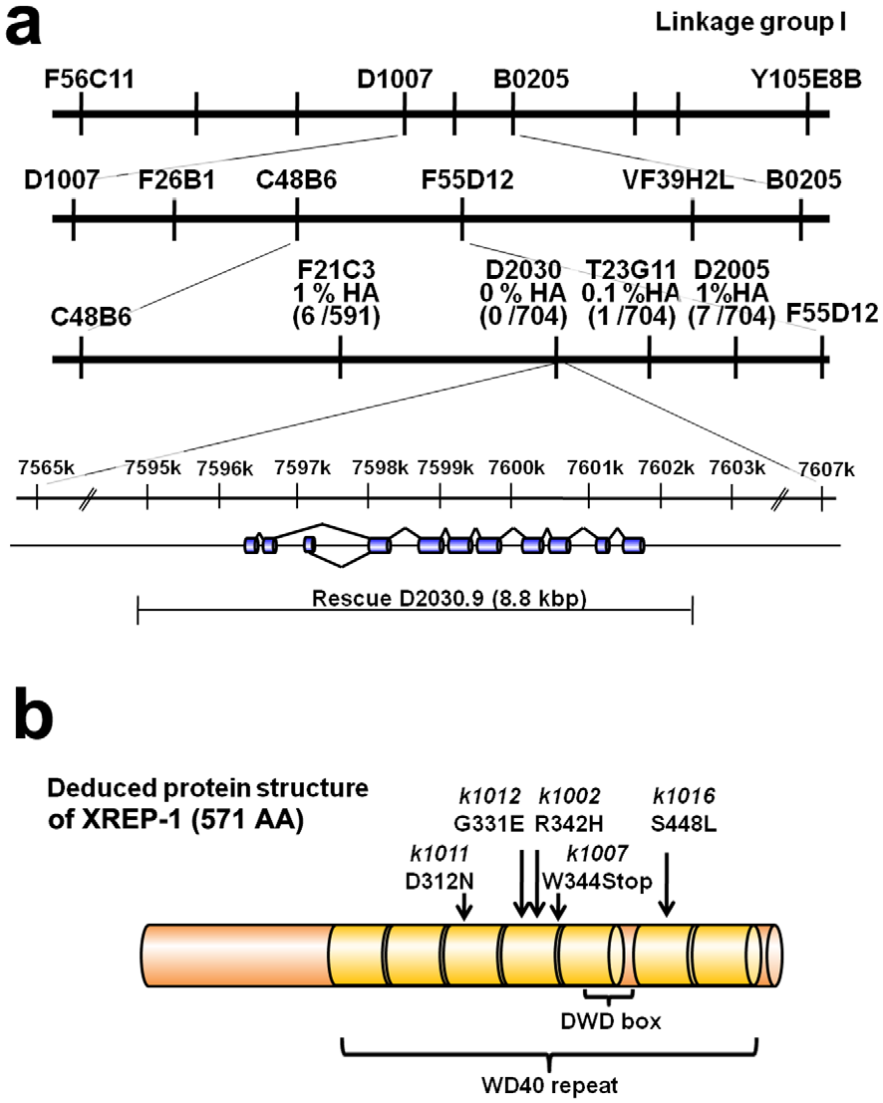
Genetic mapping and cloning of the *xrep-1* gene. (a) SNP mapping. The frequencies of Hawaiian polymorphism within F21C3, D2030, and T23G11 were 6 /591 (also designated as 1 % HA), 0 /704 (0 % HA), and 1 /704 (0.1 % HA), respectively. RNAi of D2030.9 was performed with MJCU017 by assaying the inducibility of GST-4 expression. Also, the genomic DNA fragment of D2030.9 was used to rescue the *xrep-1(k1007)* mutant phenotype. (b) Deduced protein structure of XREP-1A (see also Figure 4a-2) and locations of the five mutations identified in the present study. Amber-colored boxes represent seven WD40 domain repeats. DWD box indicates a DDB-1 (damaged DNA binding protein) WD40 binding domain. The *k1002* mutation changes CGT to CAT resulting in R to H substitution at position 342. The *k1007* mutation changes TGG to TGA resulting in W to protein chain termination at position 344. The *k1011* mutation changes GAT to AAT resulting in D to N at position 312. The *k1012* mutation changes GGA to GAA resulting in G to E at position 331. The *k1016* mutation changes TCA to TTA, resulting in S to L at position 448.

In SNP mapping involving the Hawaiian wild-type strain CB4856 crossed with the *xrep-1(k1007)* mutation, one of the 16 *xrep-1* alleles, we successfully assigned the gene *xrep-1* to the middle of LG I between the SNP markers F21C3 and T23G11. Of the total 704 recombinants analyzed, no Hawaiian polymorphism was identified within the genomic region consisting of the cosmid clone D2030, indicating that the mutation site was in or near D2030 (Fig. 2a). We amplified 12 genes within the D2030 cosmid with T7 promoter-added primers and synthesized dsRNA for soaking RNAi. RNAi of the D2030.9 gene performed in MJCU017 resulted in the induction of GST-4 expression without acrylamide (Fig. S1). Furthermore, the DNA fragment of D2030.9 amplified from N2 genomic DNA rescued the *xrep-1(k1007)* mutant phenotype (data not shown).

D2030.9 encodes WDR-23, a nematode homologue of WDR23, a member of WD40 repeat-containing proteins, which are known to exist in yeast through plants and mammals [19]; and WDR-23 was recently reported to be involved in *C. elegans* GST-4 expression [20]. Five *xrep-1* mutations sequenced so far have revealed four missense mutations and one nonsense mutation in the WD repeat domain, suggesting that this domain is important in the regulation of GST expression by keeping it from being induced in the absence of acrylamide (Fig. 2b). To avoid unnecessary confusion, we continue to use the gene name *xrep-1* and its corresponding protein name XREP-1 instead of *wdr-23* and WDR-23 unless necessary for clarification.

### GST-4 expression is not totally but partially regulated by SKN-1

**Figure 3:**
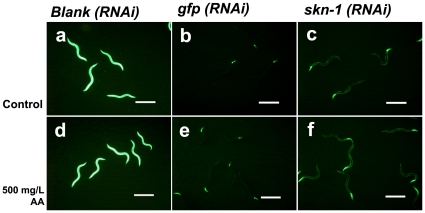
The *xrep-1*-induced GST-4 expression is partially regulated by the transcription factor SKN-1. The GST-4 expression pattern of each RNAi in the *xrep-1(k1007)* mutant, without acrylamide (control) (a-c) or treated with 500 mg/L acrylamide (d-f). (a) Strong GFP signal is detected from the whole body. (b) GST-4 expression is suppressed by *gfp(RNAi)* except for that in the pharynx. (c) GST-4 expression is suppressed by *skn-1(RNAi)* except for that in the pharynx and body-wall muscle. (d) GST-4 expression is not further induced by acrylamide over that in the animals of a. (e) GST-4 expression pattern suppressed by *gfp*(*RNAi*) is unaffected by acrylamide. (f) GST-4 expression pattern suppressed by *skn-1*(*RNAi*) is not changed by acrylamide. Scale bars, 500 µm.

We reported previously that acrylamide-induced GST-4 expression was partially regulated by the transcription factor SKN-1 [11]. To examine whether the constitutive GST expression caused by the *xrep-1* mutation was under SKN-1 control, we performed a series of knockdown experiments by feeding *C. elegans* RNAi constructs. As should be expected, the *xrep-1(k1007)* mutant constitutively emits a strong GFP signal from the whole body without acrylamide, and treating it with acrylamide did not further enhance this signal (Fig. 3). Knockdown of *gfp* resulted in the shutdown of both the constitutive and acrylamide-induced GFP signals except for that in the pharynx as expected (Fig. 3). The *skn-1* knockdown also prevented the constitutive and acrylamide-induced GST-4 expression except for that in the pharynx and in body wall muscle (Figs. 3, S2). These results suggest that not all acrylamide-induced GST-4 expression is under SKN-1 regulation.

### XREP-1 negatively regulates GST expression

Because the *xrep-1(k1007)* mutation constitutively expresses GST without acrylamide, we hypothesized that *xrep-1* negatively regulated GST-4 expression and that acrylamide triggered GST induction by preventing normal XREP-1 function. To test this hypothesis, we first constructed the *xrep-1(+)::rfp* fusion gene and transferred it into the *xrep-1(k1007)* mutant. The *xrep-1(+)::rfp* completely rescued the *xrep-1(k1007)* mutant phenotype, but XREP-1::RFP fluorescence was too weak to be useful for the present experiment (data not shown). Therefore, we used a strain, MJCU058 *{unc-119(ed3) III*, *kIs15[gst-4::rfp, gst-2::gfp, pDP#MM016B] IV}*. This strain emitted no detectable GST-4::RFP fluorescence signal, with a hardly detectable weak constitutive GST-2::GFP signal from the mouth region (Fig. S3a). Treatment of MJCU058 with acrylamide increased the RFP signal, but did not change the GFP fluorescence signal (Fig. S3b). We introduced the *xrep-1*(*k1007*) mutation into the strain MJCU058 to construct MJCU059 *{xrep-1(k1007) I; unc-119(ed3) III, kIs15 IV}*, which, similarly to the fully induced GST-4::GFP expression pattern of MJCU017, constitutively expressed a strong GST-4::RFP signal from the whole body, without changing the level of the GST-2::GFP signal (Fig. S3c).

**Figure 4:**
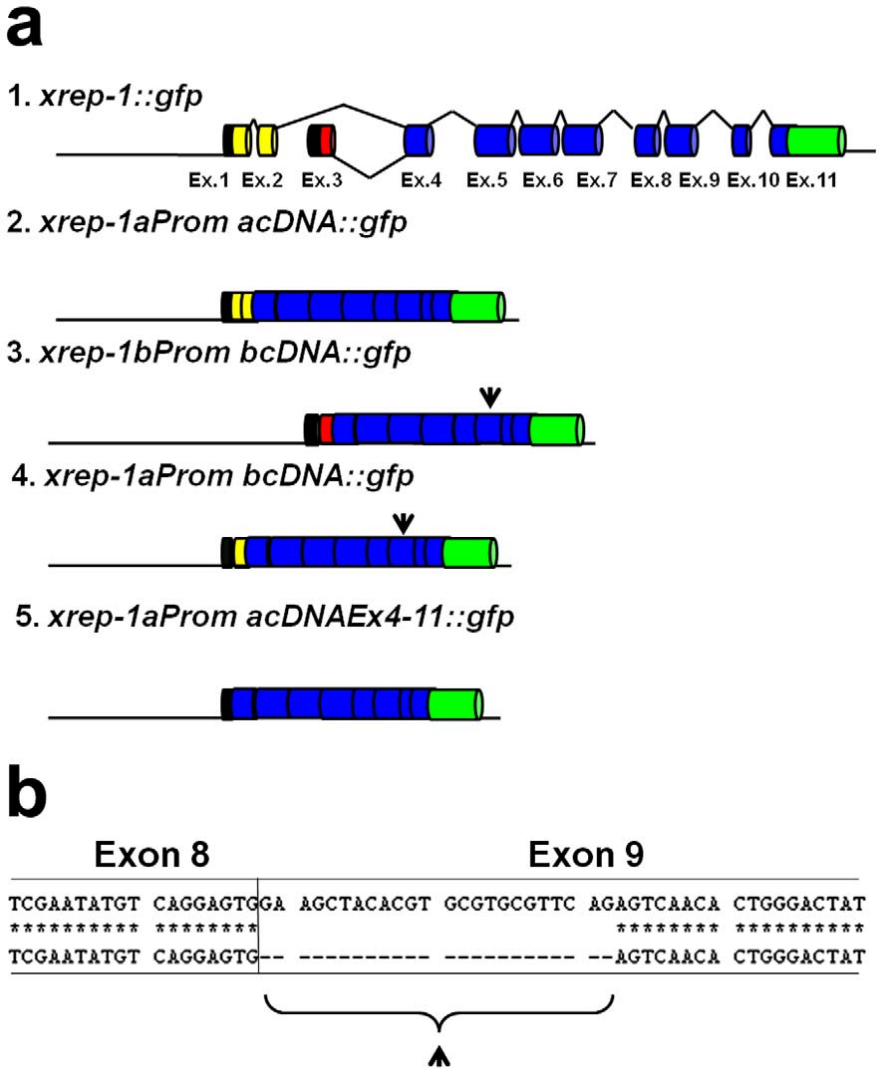
Structures of *xrep-1* fusion constructs and partial DNA sequences of *xrep-1* transcripts. (a) Structures of various *xrep-1::gfp* fusion constructs. Two yellow boxes indicate exons 1 and 2 unique to *xrep-1a*, and a red box indicates exon 3 unique to *xrep-1b*. Black boxes indicate 5’ untranslated sequences, and a green box indicates *gfp* cDNA. The arrowhead indicates a shorter exon 9, whose sequence is shown in b. (b) Partial DNA sequence alignment of *xrep-1a* cDNA (upper lines) and *xrep-1b* cDNA (lower lines). Exon 9 in *xrep-1b* cDNA was 24 bp shorter than that in *xrep-1a* (arrowhead).

**Figure 5:**
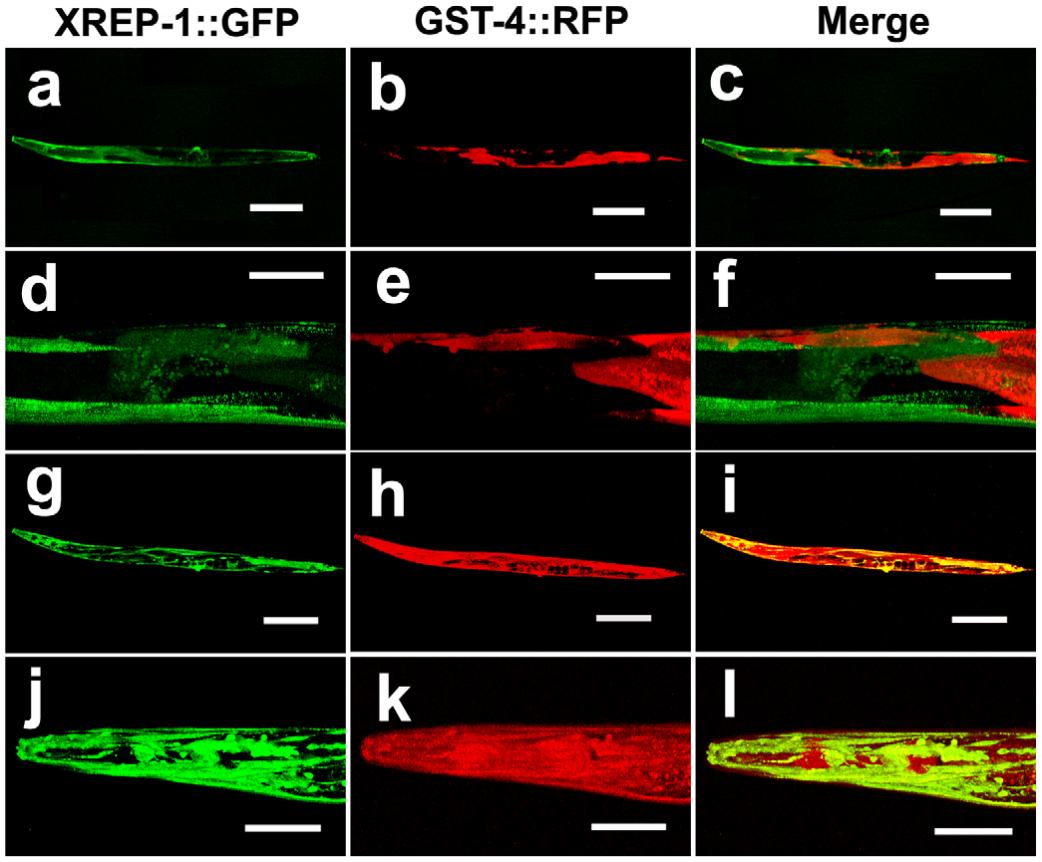
Mosaic analysis for *xrep-1* functions. (a, d) XREP-1::GFP mosaic expression pattern in MJCU080 *{kEx80[xrep-1(+)::gfp, pRF4]; xrep-1(k1007) I; unc-119(ed3) III; kIs15 IV}* without acrylamide. (b, e) GST-4::RFP expression pattern in MJCU080 without acrylamide. (c, f) Merged image. The *xrep-1(k1007)* phenotype is rescued in XREP-1::GFP-expressing cells or tissues. (g, j) XREP-1::GFP mosaic expression pattern in MJCU080, treated with 500 mg/L acrylamide. (h, k) GST-4::RFP expression pattern in MJCU080, treated with 500 mg/L acrylamide. (i, l) Merged image. GST-4::RFP expression is also detected in XREP-1::GFP-expressing cells or tissues. Scale bars, a, b, c, g, h, i, 200 µm; d, e, f, j, k, l, 50 µm. The images d, e, f, j, k, l are the respective enlargements of a, b, c, g, h, i. The images c, f, i, l are the respective merged images of a/b, d/e, g/h, j/k.

We then constructed an *xrep-1(+)::gfp* fusion gene (Fig. 4a) and introduced it into MJCU059 to obtain MJCU080 *{kEx80[xrep-1(+)::gfp, pRF4]; xrep-1(k1007) I; unc-119(ed3) III, kIs15 IV}*. The extrachromosomal array *kEx80[xrep-1(+)::gfp, pRF4]* of MJCU080 rescued the *xrep-1(k1007)* mutant phenotype and expressed GFP in the cytoplasm and nuclei of neurons, somatic gonads, intestine, and hypodermis over the whole body (Fig. 5). Because the extrachromosomal array of MJCU080 was mitotically unstable, this strain often produced animals mosaic for the *xrep-1(+)::gfp* transgene. Interestingly, although expectedly, those cells or tissues that retained the *xrep-1(+)::gfp* transgene, thus keeping the *xrep-1(k1007)* under rescue, emitted GFP signals. In contrast, cells or tissues without the *xrep-1(+)::gfp* transgene emitted no GFP signal, thus displaying the *xrep-1* mutant phenotype as expected; that is, they emitted the RFP fluorescence signal (Figs. 5a–f). After a 48-hour acrylamide treatment, however, even those cells or tissues possessing *xrep-1(+)::gfp* that emitted the GFP signal were also induced to emit RFP fluorescence (Figs. 5g–l). This mosaic analysis confirmed our earlier hypothesis that XREP-1 repressed GST from being expressed in the absence of acrylamide and de-repressed GST expression in its presence.

**Figure 6:**
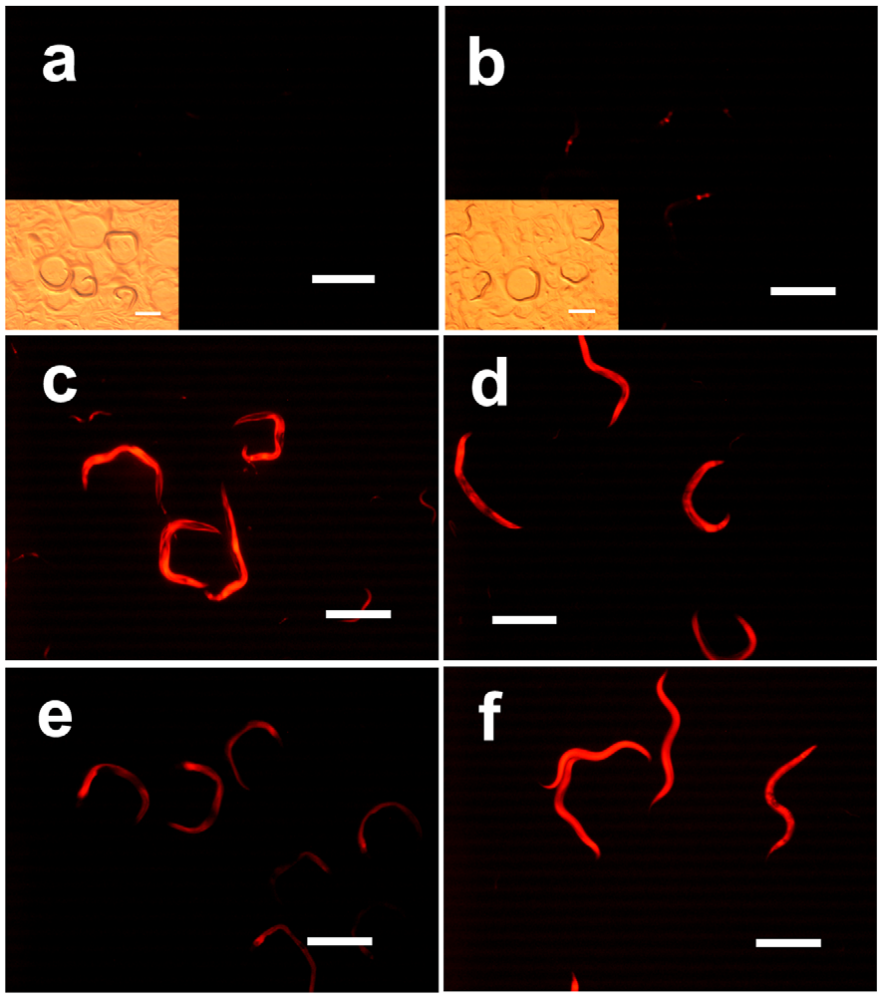
Extra copies of the *xrep-1(+)::gfp* transgene suppress acrylamide-induced GST-4::RFP expression. (a) Fluorescence plus bright field images of MJCU080 *{kEx80[xrep-1(+)::gfp, pRF4]; xrep-1(k1007) I; unc-119(ed3) III; kIs15 IV}*. Without acrylamide, no RFP signal is detected. (b) Fluorescence plus bright field images of MJCU080, treated with 500 mg/L of acrylamide for 24 hours at 20°C. Weak RFP signal is detected. (c) Fluorescence image of MJCU080, treated with 500 mg/L acrylamide for 72 hour at 20°C. RFP fluorescence signal is detected. (d) Fluorescence image of MJCU080, treated with *xrep-1(RNAi)* and 500 mg/L of acrylamide for 24 hours at 20°C. RFP fluorescence signal is detected. (e) Fluorescence image of MJCU080, treated with *xrep-1(RNAi)* for 24 hours at 20°C. RFP fluorescence signal is detected. (f) Fluorescence image of MJCU058 *{unc-119(ed3) III; kIs15 IV}*, treated with *xrep-1(RNAi)* and 500 mg/L of acrylamide for 24 hours at 20°C. RFP fluorescence signal is detected. Scale bars, 500 µm.

Notably, when MJCU080 animals were treated with acrylamide for 24 hours, the induction of GST expression was not so strong as would be expected from strains such as MJCU017 (Figs. 6a–b). Following 72 hours of acrylamide treatment, however, GST expression was well induced (Fig. 6c). We interpret the result to mean that this “super-repression” of acrylamide-induced GST expression was caused by over-expression from extra copies of the *xrep-1(+)::gfp* transgene. Its repression was eliminated when the animals were treated with *xrep-1(RNAi)* (Figs. 6d–f). This result further augments our hypothesis that XREP-1 controls GST expression through negative regulation, which is responsive to and inactivated or released by acrylamide. The XREP-1-mediated regulation of GST expression agrees with some functional evidence of this regulation: for instance, *xrep-1(k1007)* and *xrep-1(RNAi)* animals show more resistance to aldicarb than do their wild-type counterparts (manuscript in preparation).

We constructed the chromosomally-integrated stable *Is* line MJCU085 *{unc-119(ed3) III, kIs84[xrep-1(+)::gfp, pDP#MM016B]}* and confirmed that its expression pattern did not differ from that for the *Ex* line MJCU080 (Fig. S4).

### Functionality of complementary DNAs for two separate *xrep-1* gene transcripts

**Figure 7:**
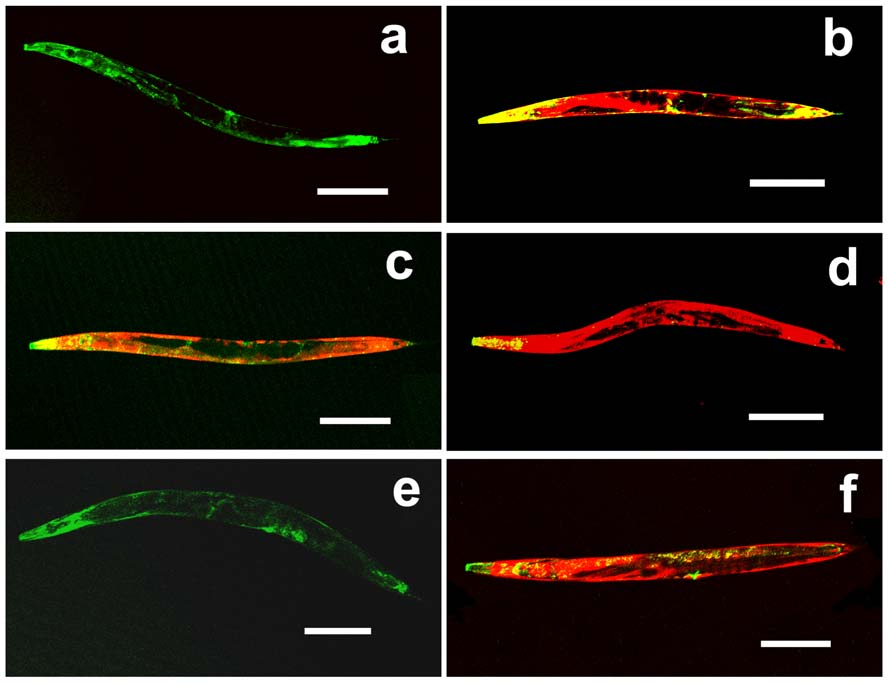
Expression patterns of *xrep-1 cDNA::gfp* fusion genes in the *xrep-1*(*k1007*) background. GFP represents XREP-1 expression and RFP indicates GST-4 expression, without (a, c, d, e) or with 500 mg/L of acrylamide (b, f). (a) Fluorescence image of MJCU086. *xrep-1aProm acDNA::gfp* completely rescues *xrep-1(k1007)* GST-4::RFP expression. (b) Fluorescence image of MJCU086. After acrylamide treatment *xrep-1aProm acDNA::gfp* induces GST-4::RFP expression normally. (c) Fluorescence image of MJCU088. *xrep-1bProm bcDNA::gfp* does not rescue *k1007* GST-4::RFP expression. (d) Fluorescence image of MJCU097. *xrep-1aProm bcDNA::gfp* does not rescue *k1007* GST-4::RFP expression. (e) Fluorescence image of MJCU091. *xrep-1aProm acDNAEx4-11::gfp* completely rescues GST-4::RFP expression. (f) Fluorescence image of MJCU091. After acrylamide treatment *xrep-1aProm acDNAEx4-11::gfp* induces GST-4::RFP expression normally. Anterior is left, scale bars 200 µm.

The *xrep-1* gene is predicted to encode a few protein isoforms according to WormBase (http://www.wormbase.org/). We obtained two transcripts from N2 animals, which were then used to synthesize *xrep-1a* and *xrep-1b* cDNAs. By sequencing them, we found that *xrep-1a* cDNA comprises 10 exons (exons 1, 2, and 4 to 11 with exclusion of exon 3) and *xrep-1b* cDNA consists of 9 exons (exons 3 to 11 with the exclusion of exons 1 and 2). Exons 4 to 11 were identical in both cDNAs except for the 9th exon; the 9th exon in *xrep-1b* cDNA was 24 bp shorter than that in *xrep-1a* (Fig. 4b). We then constructed *xrep-1aProm acDNA::gfp* and *xrep-1bProm bcDNA::gfp* fusion genes (Fig. 4a), which were used to transform MJCU059 *{xrep-1(k1007) I; unc-119(ed3); kIs15 IV}*, and obtained at least two lines of transgenic animals for each fusion gene. As observed with the *xrep-1(+)::gfp* transgene, *xrep-1aProm acDNA::gfp* expressed GST-4 in a variety of organs, tissues, or cell types in the body, such as pharynx, hypodermis, intestine, and neurons (Fig. S4), and completely rescued the *xrep-1(k1007)* mutant phenotype. GST-4 expression in the *xrep-1aProm acDNA::gfp* transgenic animal was not detected without acrylamide, but was induced with acrylamide (Figs. 7a–b). In contrast, the *xrep-1bProm bcDNA::gfp* transgene did not rescue the *xrep-1(k1007)* phenotype (Fig. 7c), as GST-4 was expressed in the absence of acrylamide (Fig. S4).

We then constructed other *gfp* fusion genes, *xrep-1aProm bcDNA::gfp* and *xrep-1aProm acDNAEx4-11::gfp* (Fig. 4a); the former was an *xrep-1b cDNA::gfp* fusion gene transcribed from the *xrep-1a* start codon, whereas the latter was an *xrep-1a cDNA::gfp* fusion gene without exons 1 through 3. Both fusion genes were then introduced into MJCU059. These fusion genes exhibited the same expression pattern as did the *xrep-1aProm acDNA::gfp* gene (Fig. S4). Also the *xrep-1aProm acDNAEx4-11::gfp* fusion gene completely rescued the *xrep-1(k1007)* phenotype (Figs. 7e–f); however, the *xrep-1aProm bcDNA::gfp* did not (Fig. 7d).

## Discussion

Of the phase II enzymes, GSTs are universally found in every organism from bacteria to humans and constitute a large family of enzymes that function as detoxifiers of both endogenous oxidative stress products and exogenous electrophilic chemical compounds [7]–[11]. Also very importantly, some GSTs are not just detoxifiers but multifunctional performers, as they participate in steroid and eicosanoid biosynthesis as well as in amino acid metabolism [9], [12], [13]. The *C. elegans* genome contains 52 *gst*-coding genes in the Alpha, Sigma, Omega, Zeta, and Pi classes (WormBase, http://www.wormbase.org/), and 18 of the GSTs were prominently up-regulated when animals were treated with acrylamide [11]. Because acrylamide is (a) found widely and abundantly in various foods as a hazardous food contaminant, (b) known as a potential carcinogen for humans, and (c) induces a large number and variety of phase II enzymes, we have selected this chemical as a model xenobiotic chemical probe that should represent a substantial number of xenobiotic compounds [11], [18], [21], [22], [23] (Fig. S5). To understand the genetic, molecular, and cellular processes of xenobiotics, we have attempted to dissect genetically a xenobiotics response pathway (*xrep*) in *C. elegans* by isolating mutants that respond abnormally to acrylamide. We have so far found four *xrep* genes: three genes (*xrep-1 I*, *-2 II*, and *-3 IV*) negatively and one gene (*xrep-4 I*) positively regulate GST expression.

The negatively regulating gene *xrep-1* encodes XREP-1, a nematode homologue of the WD-repeat containing protein 23 or WDR23. WDR exists in a broad range of organisms from yeasts to mammals as well as plants. It participates in a variety of biochemical, cellular, and organismal processes, such as signal transduction, cytoskeletal dynamics, and RNA processing [19]. A family of WDR proteins functions as a substrate adapter for ubiquitin E3 ligase, and WDR domains are predicted to form a β-propeller structure, which acts as a dock for interaction with other proteins [19]. Another family of proteins predicted to form the β-propeller structure is a group of proteins containing Kelch-repeat domains, which are also considered to serve as substrate adaptors for ubiquitin E3 ligase [19]. One such protein called Keap1 represses the bZIP transcription factor Nrf2 via the Kelch-repeat domain for degradation through the ubiquitin-proteasome pathway in the absence of oxidative or electrophilic stresses. In the presence of such stresses, Nrf2 is freed from Keap1 into the nucleus where it induces the expression of phase II enzymes [8], [24]. In *C. elegans*, the bZIP transcription factor SKN-1, which is necessary for mesendodermal differentiation during early embryogenesis [25], is found to function similarly to Nrf2 by inducing phase II enzyme expression in response to oxidative stresses [26]–[30], sodium arsenite [30], and acrylamide [11]. According to our transcriptome analysis, a large number of genes coding for such detoxifying enzymes as GSTs, UGTs, and SDRs are negatively regulated by *xrep-1* [23]. Thus, in *C. elegans*, XREP-1 might control SKN-1, similarly to the mammalian Keap1 for Nfr2 operative in the so-called antioxidant response element (ARE) pathway [9], [31]. An idea similar to this was recently reported [20].

Indeed, RNAi knockdown of *skn-1* prevented the *xrep-1*(*k1007*) mutants and acrylamide-treated animals from expressing GST-4 in most parts of the *C. elegans* body, except for the pharynx and body-wall muscle (Fig. 3). Because the RNAi knockdown of *gfp* did not prevent GST-4 expression in the pharynx, however, we assume that RNAi itself did not work in the pharynx. GST-4 expression was not further induced in the *xrep-1*(*k1007*) mutants when they were treated with acrylamide (Fig. 3), thus suggesting that all observed acrylamide-induced GST-4 expression was under control through XREP-1. SKN-1 seemed to control GST-4 expression downstream of XREP-1 in the xenobiotics response pathway, albeit in a tissue-specific fashion, as GST-4 was expressed in the body-wall muscle of *skn-1(RNAi)* animals. All five *xrep-1* mutation sites so far identified are located in the WDR domain of XREP-1 (Fig. 2). This result is consistent with the idea that this domain may be important for interaction with SKN-1.

Here we have an emerging picture of a functional similarity between the two pairs of proteins XREP-1/SKN-1 in worms and Keap1/Nrf2 in mammals, as both play a key role in the oxidative and electrophilic stress pathway. At the same time, however, this leaves us with fascinating evolutionary puzzles: (a) how the two counterparts XREP-1 and Keap1, considered as phylogenetically distant relatives (19), have converged to assume essentially an identical role in the pathway critically important in defending organisms against oxidative, xenobiotic, or other life-threatening stresses, (b) why or if really the nematode *C. elegans* lacks a homologue of the mammalian Keap1, which exists also in the insect *Drosophila* (32), and (c) why no or if any mammalian WD40 repeat proteins play a role similar to that for the *C. elegans* XREP-1.

We obtained two cDNAs, *xrep-1a* and *xrep-1b*, which were PCR products of transcripts from the N2 wild type, and analyzed their function. The *xrep-1a* cDNA consisting of the exons 4 to 11, which corresponds to a C-terminus of 775 amino acid residues, was sufficient to rescue the mutation *xrep-1(k1007)* and regulate acrylamide-induced GST expression just as does the entire *xrep-1* gene (Fig. 7). Contrarily, the *xrep-1b* cDNA showed none of these functions. In the present experiment we could not detect any other transcripts, as implicated in WormBase (http://www.wormbase.org/). Thus, we have yet to know what the *xrep-1b* or any other transcripts of the *xrep-1* gene are doing.

In summary, with the aid of *C. elegans* genetics we have so far identified four *xrep* genes that regulate the GST expression in the xenobiotics response pathway, and introduced here one of them, *xrep-1.* The gene *xrep-1* that encodes a nematode homologue of WDR23 negatively regulates a large number of phase II enzymes [23]. Currently, we are studying our three remaining *xrep* genes while continuing isolation of new Xrep mutants to understand the xenobiotics response pathways and more generally the exogenous/endogenous stress response pathways and their regulation.

## Materials and Methods

### Nematode culturing and strains

Nematode culturing and handling were carried out at 20°C as described by Brenner [33]. Strains used in this experiments were N2 (Bristol strain), CB4856 (Hawaiian polymorphic strain), CB61 *dpy-5(e61) I*, CB1091 *unc-13(e1091) I*, CB120 *unc-4(e120) II*, CB364 *dpy-18(e364) III*, DP38 *unc-119(ed3) III*, CB138 *unc-24(e138) IV*, CB270 *unc-42(e270) V*, CB678 *lon-2(e678) X*, MJCU017 *{unc-119(ed3) III, kIs17[gst-4::gfp, pDPMM#016B] X}* [11], MJCU047 *{unc-119(ed3) III, kIs41[gst-30::gfp, pDPMM#016B] X}*, and MJCU058 *{unc-119(ed3) III, kIs15[gst-4::rfp, gst-2::gfp, pDP#MM016B] IV}*. NGM plates containing 500 mg/L (about 7 mM) acrylamide were prepared as described by Hasegawa et al. [11].

### Strain construction

To make reporter constructs, all PCRs were performed with KOD Plus DNA polymerase (TOYOBO, Osaka, Japan) on N2 genomic DNA. PCR primer sequences are listed in Table S1. A fragment of *C. elegans unc-54* 3′UTR region was obtained by cutting the region with *Eco*RI and *Spe*I from the *gfp* vector pPD95.77 (kindly provided by A. Fire, Stanford University) and integrated into the equivalent restriction site of pDsRed-Monomer (Clontech, CA, USA) for *rfp* (red fluorescence protein) vector pMH06.12-Red. PCR primers for *gst-2*, *gst-4*, and *gst-30* were designed to amplify predicted promoters for each gene, about 1.2–0.8 kbp upstream from the predicted start sites spanning over full coding regions without the stop codons. PCR-amplified DNA fragments were digested with the appropriate restriction enzymes (Table S1) and ligated into the *gfp* vector pPD95.77 (for *gst-2::gfp* and *gst-30::gfp*) or the *rfp* vector pMH06.12-Red (for *gst-4::rfp*). Each reporter construct (100 µg/mL) so obtained was co-injected with an equal concentration of pDP#MM016B into the gonadal arms of *unc-119(ed3)* adult hermaphrodites [34], [35] to obtain MJCU013 *{unc-119(ed3) III; kEx13[gst-4::rfp, gst-2::gfp, pDP#MM016B]}* and MJCU024 *{unc-119(ed3) III; kEx24[gst-30::gfp, pDP#MM016B]}*. Each transgene's extrachromosomal array was chromosomally integrated by the method of Mitani [36], and transgenic animals thus obtained were outcrossed at least three times with N2 to obtain MJCU058 *{unc-119(ed3) III; kIs15[gst-4::rfp, gst-2::gfp, pDP#MM016B] IV}* and MJCU047 *{unc-119(ed3) III; kIs41[gst-30::gfp, pDPMM#016B] X}*. Linkage mapping with the conventional markers (listed in the sub-section Nematode culturing and strains) located the fusion gene *gst-4::rfp, gst-2::gfp* of MJCU058 integrated in the linkage group IV and, similarly, the fusion gene *gst-30::gfp* of MJCU047 in X (data not shown). By the standard genetic methods, *xrep-1*(*k1007*) was transferred into MJCU058 *{unc-119(ed3) III; kIs15 IV}* to construct MJCU059 *{xrep-1(k1007) I; unc-119(ed3) III; kIs15 IV}*. Fluorescence expression patterns were observed with a Nikon SMZ800 dissection microscope equipped with a fluorescence filter.

### Mutant isolation and mapping

We mutagenized the transgenic strains MJCU017 and MJCU047 with 50 mM ethyl methanesulfonate (EMS), following essentially the method described by Brenner [33] to obtain mutants expressing GST abnormally. Twenty-four mutants obtained independently were outcrossed at least three times with N2 wild type to reduce extraneous mutations. By complementation testing, 16 of 24 mutants were grouped as the same gene *xrep-1*. The allele *xrep-1(k1007)* was first mapped to linkage group I by using conventional markers, *dpy-5(e61) I*, *unc-4(e120) II*, *dpy-18(e364) III*, *unc-24(e138) IV*, *unc-42(e270) V*, *lon-2(e678) X*, and their positions were then narrowed by single-nucleotide polymorphism mapping [37]. We defined a genomic region between the SNP markers F21C3 and T23G11. No Hawaiian polymorphism was found within D2030 (total 704 recombinants inquired) indicating that the mutation site was nearby or within the cosmid clone D2030. We amplified 12 genes within D2030 with T7 promoter-added primers and synthesized dsRNA for soaking RNAi [38]. From the soaking RNAi results, the candidate gene D2030.9 was selected, PCR-amplified from the N2 wild-type genomic DNA, and co-injected with the pRF4 marker DNA into mutant animals following the method by Mello et al. [34].

### *xrep-1::gfp* fusion gene construction

Five different *gfp* fusion genes, *xrep-1(+)::gfp*, *xrep-1aProm acDNA::gfp*, *xrep-1bProm bcDNA::gfp*, *xrep-1aProm bcDNA::gfp*, and *xrep-1aProm acDNAEx4-11::gfp*, were constructed. PCR primer sequences are listed in Table S2. The predicted *xrep-1* promoter (2,110 bp upstream from the predicted start site) plus the deduced coding region was amplified with the primers XREP-1abp_HindIII_For and XREP-1ab_BamHI_Rev (Table S2) by PCR from the N2 genomic DNA and ligated into the pPD95.77 *gfp* expression vector to obtain the *xrep-1(+)::gfp* construct. Two isoforms of *xrep-1* cDNA, *xrep-1a* and *xrep-1b*, were each amplified with the primers XREP-1ap_cDNA_For and XREP-1ab_BamHI_Rev (*xrep-1a* cDNA) or XREP-1bp_cDNA_For and XREP-1ab_BamHI_Rev (*xrep-1b* cDNA) (Table S2) by PCR from the two isoformic N2 cDNAs as templates originally derived from two respective transcripts. The *xrep-1a* or *xrep-1b* promoter was amplified with the primers XREP-1abp_HindIII_For and XREP-1ap_cDNA_Rev (*xrep-1a* promoter) or XREP-1abp_HindIII_For and XREP-1bp_cDNA_Rev (*xrep-1b* promoter) (Table S2) by PCR from the N2 genomic DNA. Either *xrep-1a* promoter and *xrep-1a* cDNA or *xrep-1b* promoter and *xrep-1b* cDNA were connected and ligated into the pPD95.77 *gfp* expression vector to obtain the *xrep-1aProm acDNA::gfp* or *xrep-1bProm bcDNA::gfp* construct (Figure 4a). Next, *xrep-1b* cDNA, connected to the *xrep-1a* promoter, was ligated into the pPD95.77 to obtain *xrep-1aProm bcDNA::gfp* (Figure 4a).

Furthermore, *xrep-1a* cDNA containing only exons 4 to 11 was amplified with the primers XREP-1ap_Exon4-11_For and XREP-1ab_BamHI_Rev (Table S2) from the N2 cDNA derived from the *xrep-1a* transcript. Again the *xrep-1a* promoter was amplified with primers XREP-1abp_HindIII_For and XREP-1ap_Exon4-11_Rev (Table S2) from the N2 genomic DNA and connected with the *xrep-1a* cDNA exon 4 to 11 and ligated into the pPD95.77 *gfp* expression vector to obtain *xrep-1aProm acDNAEx4-11::gfp* (Figure 4a). One hundred µg/mL of each reporter construct so obtained was co-injected with an equal concentration of pDP#MM016B or pRF4 into the gonadal arms of *unc-119*(*ed3*) or MJCU059 *{xrep-1(k1007) I; unc-119(ed3) III; kIs15 IV}* adult hermaphrodites as described [34], [35]. Integration of transgenic extrachromosomal arrays into chromosomes was performed as described by Mitani [36], and integrated lines were outcrossed two times with N2 wild type. Transgenic animals so obtained were MJCU080 *{xrep-1(k1007) I; unc-119(ed3) III; kIs15 IV; kEx80[xrep-1(+)::gfp, pRF4]}*, MJCU081 *{xrep-1(k1007) I; unc-119(ed3) III; kIs15 IV; kEx81[xrep-1(+)::gfp, pRF4]}*, MJCU085 *{unc-119(ed3) III; kIs84[xrep-1(+)::gfp, pDP#MM016B]}*, MJCU086 *{xrep-1(k1007) I; unc-119(ed3) III; kIs15 IV; kEx86[xrep-1aProm acDNA::gfp, pRF4]}*, MJCU087 *{xrep-1(k1007) I; unc-119(ed3) III; kIs15 IV; kEx87[xrep-1aProm acDNA::gfp, pRF4]}*, MJCU088 *{xrep-1(k1007) I; unc-119(ed3) III; kIs15 IV; kEx88[xrep-1bProm bcDNA::gfp, pRF4]}*, MJCU089 *{xrep-1(k1007) I; unc-119(ed3) III; kIs15 IV; kEx89[xrep-1bProm bcDNA::gfp, pRF4]}*, MJCU091 *{xrep-1(k1007) I; unc-119(ed3) III; kIs15 IV; kEx91[xrep-1aProm acDNAEx4-11::gfp, pRF4]}*, MJCU094 *{xrep-1(k1007) I; unc-119(ed3) III; kIs15 IV; kEx94[xrep-1aProm acDNAEx4-11::gfp, pRF4]}*, MJCU097 *{xrep-1(k1007) I; unc-119(ed3) III; kIs15 IV; kEx97[xrep-1aPromb cDNA::gfp, pRF4]}*, and MJCU098 *{xrep-1(k1007) I; unc-119(ed3) III; kIs15 IV; kEx98[xrep-1a Prom bcDNA::gfp, pRF4]}*.

### RNAi

Gene fragments of *skn-1* cDNA, *xrep-1* cDNAEx4-11, or *gfp* were prepared by PCR amplification of *C. elegans* N2 cDNA, genomic DNA, or plasmid vector pPD95.77, respectively, with primers (Table S3). Each PCR fragment was digested with *Eco*RI and cloned into the *Eco*RI restriction site of the RNAi vector pPD129.36 (kindly provided by A. Fire, Stanford University). The PCR fragment-ligated plasmid or the blank vector pPD129.36 was used to transform *E. coli* HT115 [39].

For RNAi experiments, synchronized L1-stage animals were first cultured for 48 hours at 20°C on NGM (containing 50 µg/mL ampicillin and 12.5 µg/mL tetracycline) plates seeded with *E. coli* HT115 transformed with each different RNAi plasmid. The animals were then collected and transferred onto NGM plates with or without 500 mg/L acrylamide, seeded with each different *E. coli* HT115 RNAi bacteria. After 24 to 48 hours of incubation, the animals were observed for GFP expression with both a Nikon SMZ800 dissection microscope equipped with a fluorescence filter and a ZEISS Axiovert 200 microscope equipped with a confocal laser-scanning module.

## Supporting Information

Figure S1 GST-4 expression is induced when the transgenic MJCU017 animals are treated with soaking RNAi of D2020.9 (*xrep-1*). (a) MJCU017 animals treated with blank RNAi. No GST-4::GFP expression is detected (arrows point to three animals). (b) MJCU017 animals treated with *xrep-1(RNAi)*. GST-4::GFP expression is induced. Scale bar, 500 µm.
(1.29 MB TIF)

Figure S2 GST-4 expression induced by acrylamide and *xrep-1(k1007)* mutation is prevented by *skn-1(RNAi)* except for that in the pharynx and body-wall muscle. (a-c) GST-4 expression patterns in MJCU017 treated with 500 mg/L acrylamide. (d-f) GST-4 expression patterns in *xrep-1(k1007)* without acrylamide. (g-i) GST-4 expression patterns of *skn-1(RNAi)* in *xrep-1(k1007)* without acrylamide. Scale bars, 50 µm.
(1.33 MB TIF)

Figure S3 (a) Fluorescence plus bright field images of MJCU058 *{unc-119(ed3) III; kIs15[gst-4::rfp, gst-2::gfp, pDP#MM016B] IV}*. Without acrylamide, no fluorescence signal is detected. (b) Fluorescence image of MJCU058, treated with 500 mg/L of acrylamide at 20°C for 24 hours. RFP fluorescence signal is detected. (c) Fluorescence image of MJCU059 *{xrep-1(k1007) I; unc-119(ed3) III; kIs15 IV}*. RFP fluorescence signal is detected. (d-f) RFP fluorescence signal in the MJCU058 *{unc-119(ed3) III; kIs15 IV}* animal, treated with 500 mg/L of acrylamide at 20°C for 24 hours. (d) Head. (e) Vulva. (f) Tail. Scale bars, a-c, 500 µm; d-f, 50 µm.
(2.14 MB TIF)

Figure S4 Expression patterns of various *xrep-1::gfp* fusion genes. (a-c) XREP-1::GFP expression. (d-f) XREP-1aProm acDNA::GFP expression. (g-i) XREP-1bProm bcDNA::GFP expression patterns. (j-l) XREP-1aProm bcDNA::GFP expression. (m-o) XREP-1aProm acDNAEx4-11::GFP expression. Scale bars, 50 µm.
(1.79 MB TIF)

Figure S5 GST and UGT responses in transgenic animals against several xenobiotics. Young adult transgenics were transferred into NGM plates containing each xenobiotic and incubated at 25°C for 24 hours. Transgenic animals used in these experiments were MJCU017 *{unc-119(ed3) III; kIs17[gst-4::gfp, pDP#MM016B] X}*, MJCU028 *{unc-119(ed3) III; kEx28[gsto-2b::cfp, pDP#MM016B]}* (23), MJCU003 *{kEx3[gst-7::gfp, pRF4]}* (11), MJCU047 *{unc-119(ed3) III; kIs41[gst-30::gfp, pDP#MM016B] X}*, and MJCU050 *{unc-119(ed3) III; kIs20[ugt-13p::gfp, pDP#MM016B] III}* (21). Control, without xenobiotics; MeHg (500 nM Methylmercury); Paraquat (20 mM Paraquat); tBOOH (1 mM *tert*-Butyl hydroperoxide); AA (7 mM Acrylamide). (a) GST-4::GFP expression was not detected. Arrows indicate animals. (b) GST-4::GFP expression was induced when animals were treated with MeHg. (c) GST-4::GFP expression was slightly induced (arrows) when animals were treated with paraquat. (d) GST-4::GFP expression was induced when animals were treated with tBOOH. (f) Weak GSTO-2B::CFP expression was detected. (g-i) GSTO-2B::CFP expression was induced when animals were treated with MeHg, paraquat, and tBOOH. (k) Weak GST-7::GFP expression was detected. (l-n) GST-7::GFP expression was induced when animals were treated with MeHg, paraquat, and tBOOH. (p) GST-30::GFP expression was not detected. Arrows indicate animals. (q) GST-30::GFP expression was induced when animals were treated with MeHg (arrows). (r) GST-30::GFP expression was not induced when animals (arrows) were treated with paraquat. (s) GST-30::GFP expression was induced when animals were treated with tBOOH (arrows). (u) Weak UGT-13::GFP expression was detected. (v-x) UGT-13::GFP expression was induced when animals were treated with MeHg, paraquat, and tBOOH. (e, j, o, t, y) All of the GST- and UGT-fused GFP expressions were strongly induced when animals were treated with AA (11). Scale bars, 500 µm.
(1.64 MB TIF)

Table S1 Primers for *gst::reporter* fusion genes.
(0.02 MB DOC)

Table S2 Primers for *xrep-1::reporter* fusion genes.
(0.02 MB DOC)

Table S3 Primers for RNAi.
(0.02 MB DOC)
