# Supplementary figures and images for: Genetic and Cellular Characterization of Caenorhabditis elegans Mutants Abnormal in the Regulation of Many Phase II Enzymes
Source: PLoS One. 2010 Jun 17;5(6):e11194. doi: 10.1371/journal.pone.0011194 (PMC2887452; doi:10.1371/journal.pone.0011194)

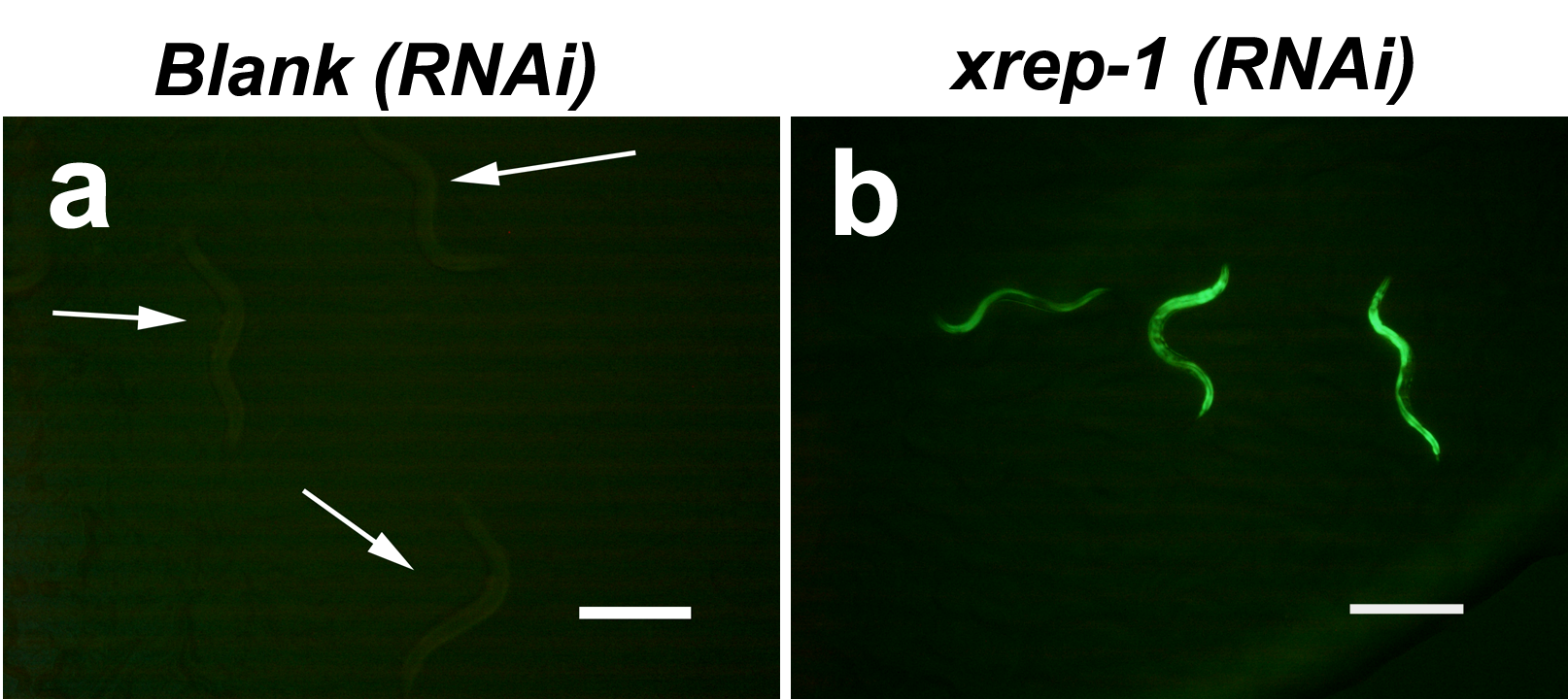

Supplement: Figure S1 — GST-4 expression is induced when the transgenic MJCU017 animals are treated with soaking RNAi of D2020.9 (xrep-1). (a) MJCU017 animals treated with blank RNAi. No GST-4::GFP expression is detected (arrows point to three animals). (b) MJCU017 animals treated with xrep-1(RNAi). GST-4::GFP expression is induced. Scale bar, 500 µm. (1.29 MB TIF) [file pone.0011194.s001.tif]

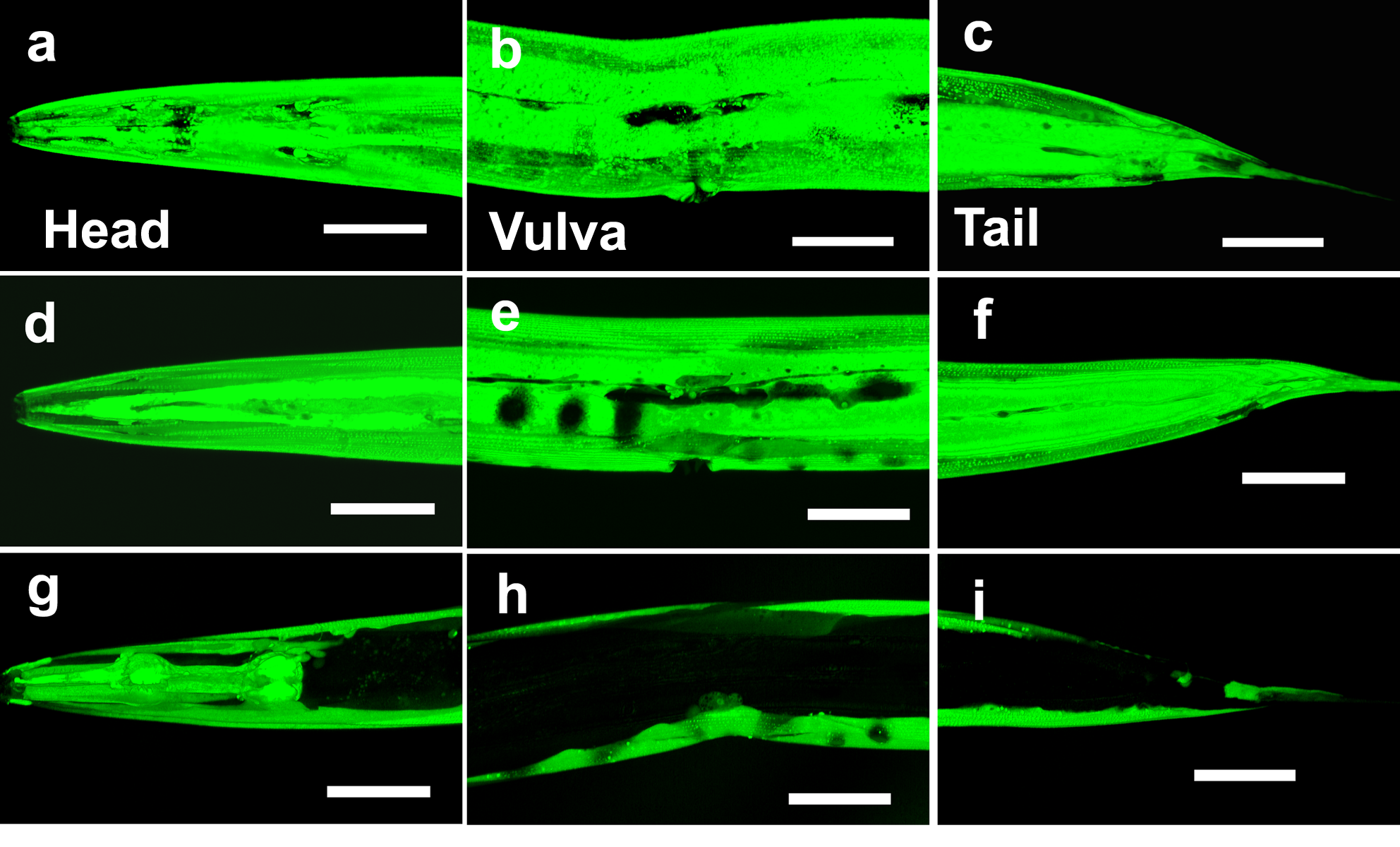

Supplement: Figure S2 — GST-4 expression induced by acrylamide and xrep-1(k1007) mutation is prevented by skn-1(RNAi) except for that in the pharynx and body-wall muscle. (a-c) GST-4 expression patterns in MJCU017 treated with 500 mg/L acrylamide. (d-f) GST-4 expression patterns in xrep-1(k1007) without acrylamide. (g-i) GST-4 expression patterns of skn-1(RNAi) in xrep-1(k1007) without acrylamide. Scale bars, 50 µm. (1.33 MB TIF) [file pone.0011194.s002.tif]

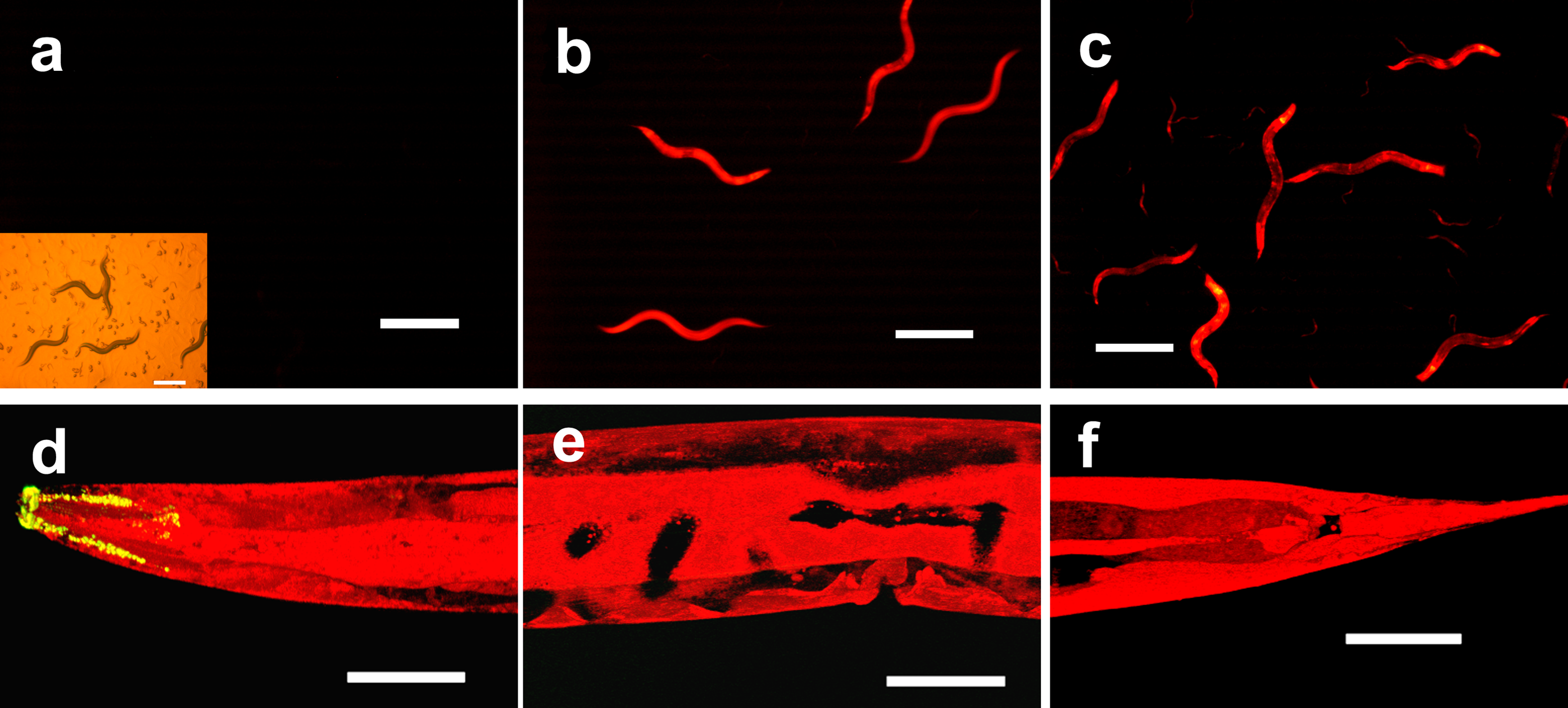

Supplement: Figure S3 — (a) Fluorescence plus bright field images of MJCU058 {unc-119(ed3) III; kIs15[gst-4::rfp, gst-2::gfp, pDP#MM016B] IV}. Without acrylamide, no fluorescence signal is detected. (b) Fluorescence image of MJCU058, treated with 500 mg/L of acrylamide at 20°C for 24 hours. RFP fluorescence signal is detected. (c) Fluorescence image of MJCU059 {xrep-1(k1007) I; unc-119(ed3) III; kIs15 IV}. RFP fluorescence signal is detected. (d-f) RFP fluorescence signal in the MJCU058 {unc-119(ed3) III; kIs15 IV} animal, treated with 500 mg/L of acrylamide at 20°C for 24 hours. (d) Head. (e) Vulva. (f) Tail. Scale bars, a-c, 500 µm; d-f, 50 µm. (2.14 MB TIF) [file pone.0011194.s003.tif]

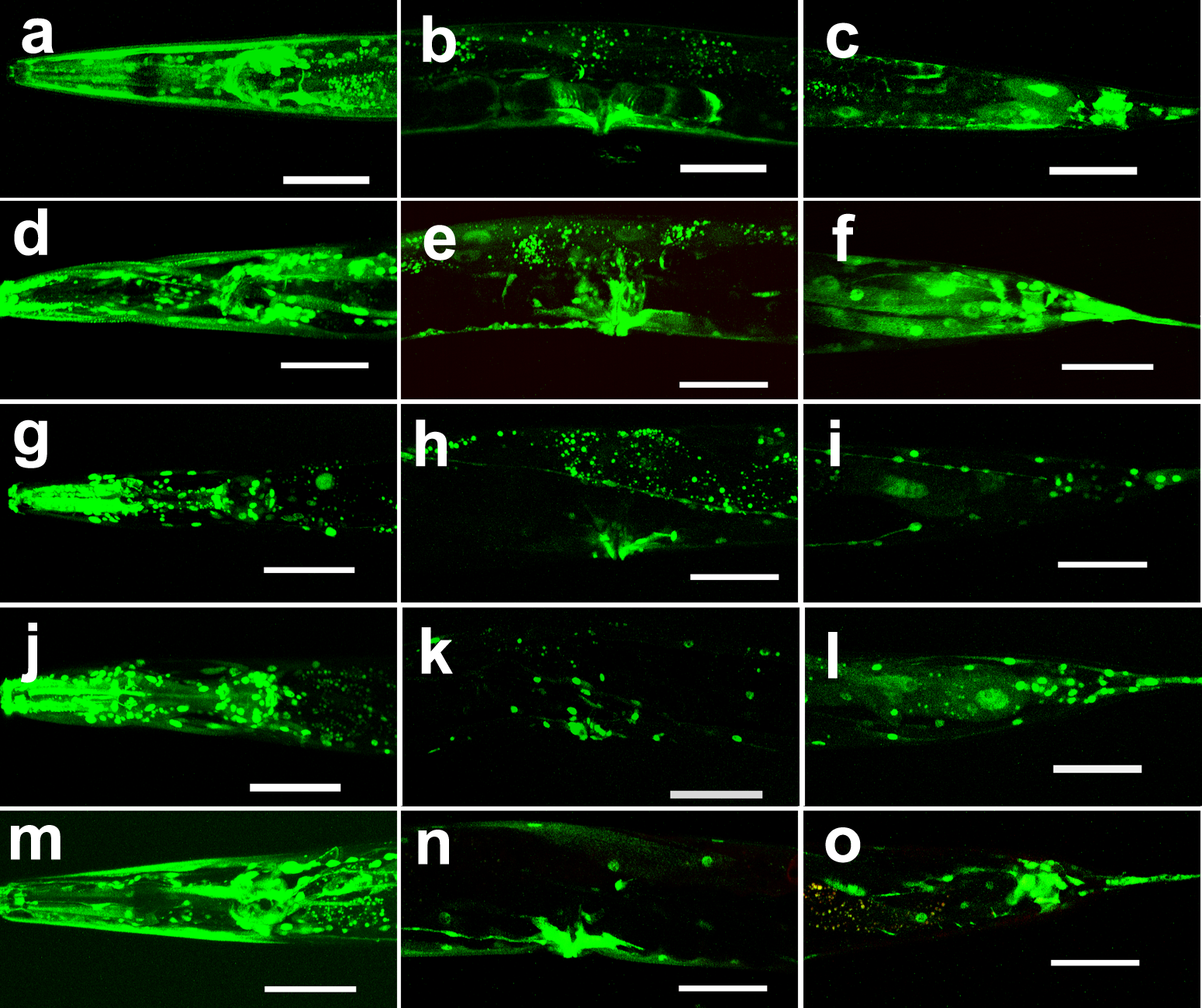

Supplement: Figure S4 — Expression patterns of various xrep-1::gfp fusion genes. (a-c) XREP-1::GFP expression. (d-f) XREP-1aProm acDNA::GFP expression. (g-i) XREP-1bProm bcDNA::GFP expression patterns. (j-l) XREP-1aProm bcDNA::GFP expression. (m-o) XREP-1aProm acDNAEx4-11::GFP expression. Scale bars, 50 µm. (1.79 MB TIF) [file pone.0011194.s004.tif]

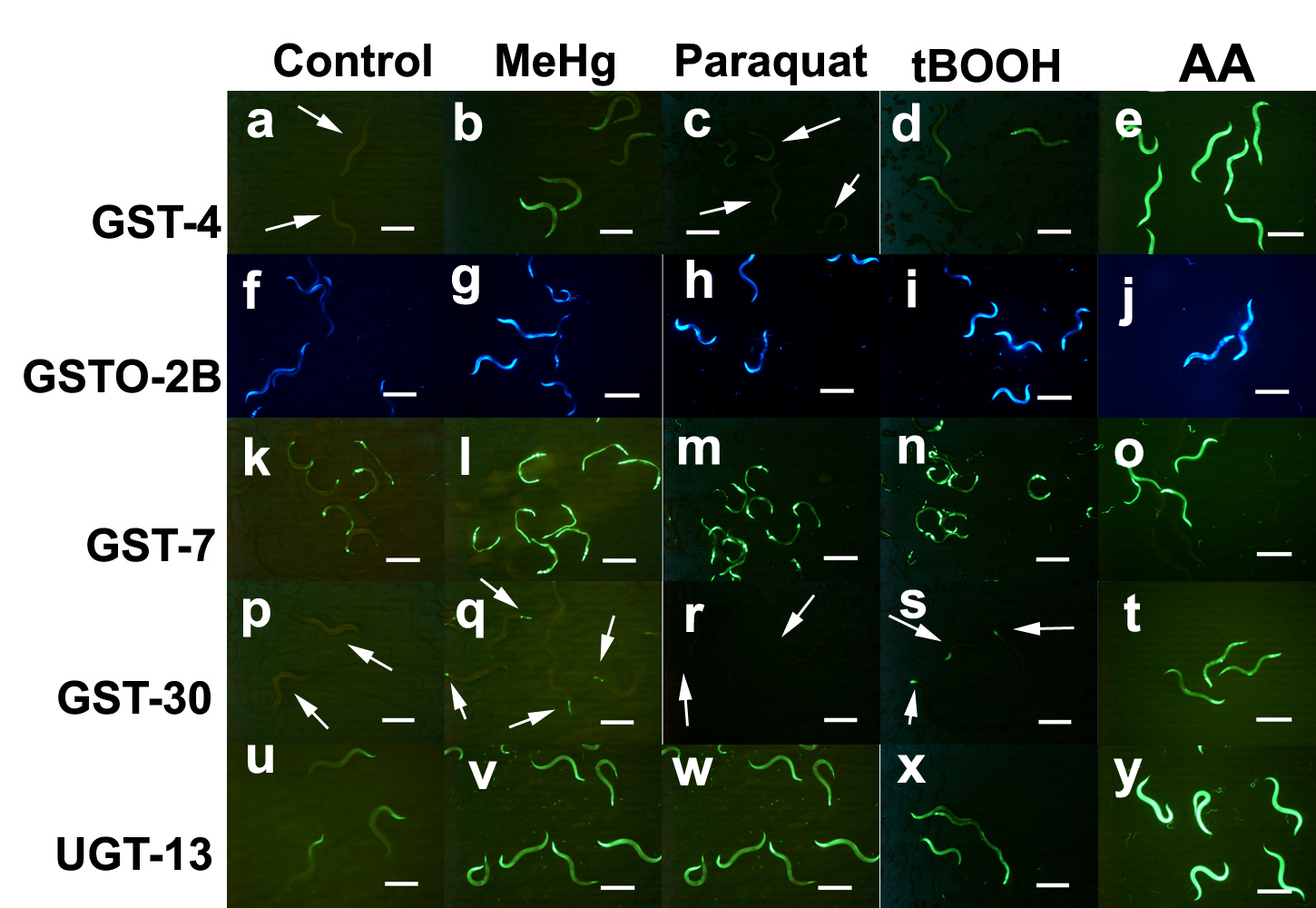

Supplement: Figure S5 — GST and UGT responses in transgenic animals against several xenobiotics. Young adult transgenics were transferred into NGM plates containing each xenobiotic and incubated at 25°C for 24 hours. Transgenic animals used in these experiments were MJCU017 {unc-119(ed3) III; kIs17[gst-4::gfp, pDP#MM016B] X}, MJCU028 {unc-119(ed3) III; kEx28[gsto-2b::cfp, pDP#MM016B]} (23), MJCU003 {kEx3[gst-7::gfp, pRF4]} (11), MJCU047 {unc-119(ed3) III; kIs41[gst-30::gfp, pDP#MM016B] X}, and MJCU050 {unc-119(ed3) III; kIs20[ugt-13p::gfp, pDP#MM016B] III} (21). Control, without xenobiotics; MeHg (500 nM Methylmercury); Paraquat (20 mM Paraquat); tBOOH (1 mM tert-Butyl hydroperoxide); AA (7 mM Acrylamide). (a) GST-4::GFP expression was not detected. Arrows indicate animals. (b) GST-4::GFP expression was induced when animals were treated with MeHg. (c) GST-4::GFP expression was slightly induced (arrows) when animals were treated with paraquat. (d) GST-4::GFP expression was induced when animals were treated with tBOOH. (f) Weak GSTO-2B::CFP expression was detected. (g-i) GSTO-2B::CFP expression was induced when animals were treated with MeHg, paraquat, and tBOOH. (k) Weak GST-7::GFP expression was detected. (l-n) GST-7::GFP expression was induced when animals were treated with MeHg, paraquat, and tBOOH. (p) GST-30::GFP expression was not detected. Arrows indicate animals. (q) GST-30::GFP expression was induced when animals were treated with MeHg (arrows). (r) GST-30::GFP expression was not induced when animals (arrows) were treated with paraquat. (s) GST-30::GFP expression was induced when animals were treated with tBOOH (arrows). (u) Weak UGT-13::GFP expression was detected. (v-x) UGT-13::GFP expression was induced when animals were treated with MeHg, paraquat, and tBOOH. (e, j, o, t, y) All of the GST- and UGT-fused GFP expressions were strongly induced when animals were treated with AA (11). Scale bars, 500 µm. (1.64 MB TIF) [file pone.0011194.s005.tif]
