# Supplementary material for: Genetic and Cellular Characterization of Caenorhabditis elegans Mutants Abnormal in the Regulation of Many Phase II Enzymes
Source: PLoS One. 2010 Jun 17;5(6):e11194. doi: 10.1371/journal.pone.0011194 (PMC2887452; doi:10.1371/journal.pone.0011194)
Supplement: Table S1 — Primers for gst::reporter fusion genes. (0.02 MB DOC) [file pone.0011194.s006.doc]

**Table S1 Primers for *gst*::*reporter* fusion genes**

GST-4_Ver3_SalI_For, 5’- GGGTCGACTTTTGCAGACTAAAAATAACTACTCTG -3’

GST-4_BamH1_Red_Rev, 5’- GGGGATCC**GG**AACAATACTATCCTTTCTTGTTGCC -3’

GST-2_Ver2_HindIII_For, 5’- CGAAGCTTCCAGCGAGTCCTAGATGTTTAAA -3’

GST-2_BamHI_Rev, 5’- GGGGATCCCAATTTTCTGATAAAAACATTCAAATG -3’

GST-30_PstI_For, 5’- AATTCTGCAGGTGACACTCTCTTCCATGCTTTTCA -3’

GST-30_BamH1_Rev, 5’- GGGGATCCAAATGGTGTAACTGGACGAGTCTCAAC -3’
