# Supplementary material for: Genetic and Cellular Characterization of Caenorhabditis elegans Mutants Abnormal in the Regulation of Many Phase II Enzymes
Source: PLoS One. 2010 Jun 17;5(6):e11194. doi: 10.1371/journal.pone.0011194 (PMC2887452; doi:10.1371/journal.pone.0011194)
Supplement: Table S2 — Primers for xrep-1::reporter fusion genes. (0.02 MB DOC) [file pone.0011194.s007.doc]

**Table S2 Primers for *xrep-1*::*reporter* fusion genes**

XREP-1abp_HindIII_For, 5’- CCCGGAAGCTTCTCCGGATCTCGTCACGAATAT -3’

XREP-1ap_cDNA_Rev, 5’- CAGTTGCCCATTCGAGCATAGGGCGAAGCACTGTA -3’

XREP-1ap_cDNA_For, 5’- CTATGCTCGAATGGGCAACTGGATAACGTCGACGT -3’

XREP-1ab_BamHI_Rev, 5’- GGCGGATCCATTTTGAGAGATGCTGCTCGATGAGC -3’

XREP-1bp_cDNA_Rev, 5’- TTTGTAAGGCATAGACGTTGAATACGAAATGAAGA -3’

XREP-1bp_cDNA_For, 5’- TTCAACGTCTATGCCTTACAAAAGACATTCCTCTT -3’

XREP-1ap_Exon4-11_Rev, 5’- CATTGCTGTGATTCATTCGAGCATAGGGCGAAGCA -3’

XREP-1ap_Exon4-11_For, 5’- TATGCTCGAATGAATCACAGCAATGACAGTGACAC -3’
