# Supplementary material for: Genetic and Cellular Characterization of Caenorhabditis elegans Mutants Abnormal in the Regulation of Many Phase II Enzymes
Source: PLoS One. 2010 Jun 17;5(6):e11194. doi: 10.1371/journal.pone.0011194 (PMC2887452; doi:10.1371/journal.pone.0011194)
Supplement: Table S3 — Primers for RNAi. (0.02 MB DOC) [file pone.0011194.s008.doc]

**Table S3 Primers for RNAi**

CeSKN-1_EcoRI_For, 5’- GGAATTCGGCCAATCCAAATATGATTATCCA -3’

CeSKN-1_EcoRI_Rev, 5’- GGAATTCGGGCAGCAACCTTGTTCTTTCCG -3’

XREP-1_Exon4-11_For, 5’- AGGGAACAACATATTGCATTTAGT-3’

XREP-1_Exon4-11_EcoRI_Rev, 5’- GGGAATTCTTGGGATGATCGTATGGTGCAA -3’

GFP_EcoRI_For, 5’- GGGAATTCACTGGAGTTGTCCCAATTCTT -3’

GFP_EcoRI_Rev, 5’- GGGAATTCATCCATGCCATGTGTAATCCC -3’
